# Evolving Catalytic Properties of the MLL Family SET Domain

**DOI:** 10.1016/j.str.2015.07.018

**Published:** 2015-10-06

**Authors:** Ying Zhang, Anshumali Mittal, James Reid, Stephanie Reich, Steven J. Gamblin, Jon R. Wilson

**Affiliations:** 1 The Francis Crick Institute, Mill Hill Laboratory, London NW7 1AA, UK; 2 Domainex, Cambridge CB4 0GH, UK

## Abstract

Methylation of histone H3 lysine-4 is a hallmark of chromatin associated with active gene expression. The activity of H3K4-specific modification enzymes, in higher eukaryotes the MLL (or KMT2) family, is tightly regulated. The MLL family has six members, each with a specialized function. All contain a catalytic SET domain that associates with a core multiprotein complex for activation. These SET domains segregate into three classes that correlate with the arrangement of targeting domains that populate the rest of the protein. Here we show that, unlike MLL1, the MLL4 SET domain retains significant activity without the core complex. We also present the crystal structure of an inactive MLL4-tagged SET domain construct and describe conformational changes that account for MLL4 intrinsic activity. Finally, our structure explains how the MLL SET domains are able to add multiple methyl groups to the target lysine, despite having the sequence characteristics of a classical monomethylase.

## Highlights

• MLL4 SET domain has significant H3K4 methyltransferase activity without WRAD
• MLL4 SET domain structure (inactivated by tag) reveals differences to MLL1
• The postSET loop region contributes to the intrinsic methyltransferase activity
• MLL4 can catalyze multiple methylations on H3K4 but prefers monomethylation

## Graphical Abstract

**Figure:**
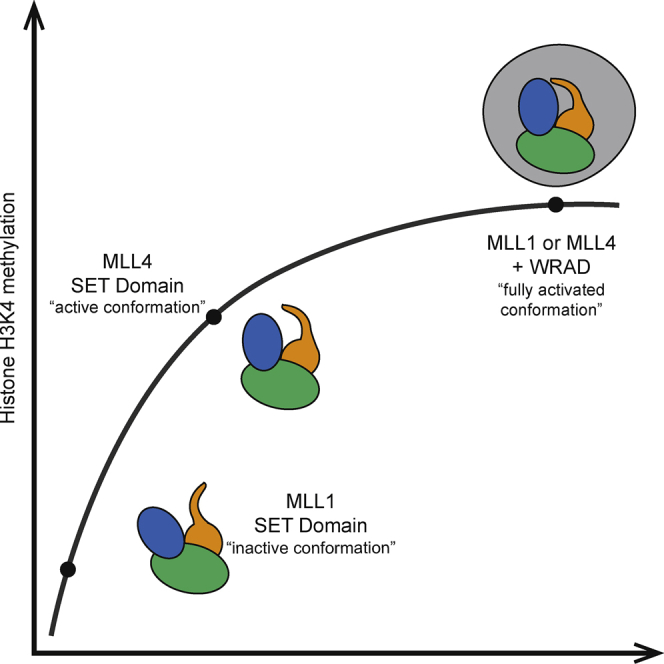

## Teaser

The MLL family of histone H3K4-specific methyltransferases contains six members, each with a unique role. Zhang et al. show that, unlike MLL1, the MLL4 SET domain has high intrinsic activity without the cognate complex. The authors have identified a new structural element that contributes to this intrinsic activation.

## Introduction

**Figure 1:**
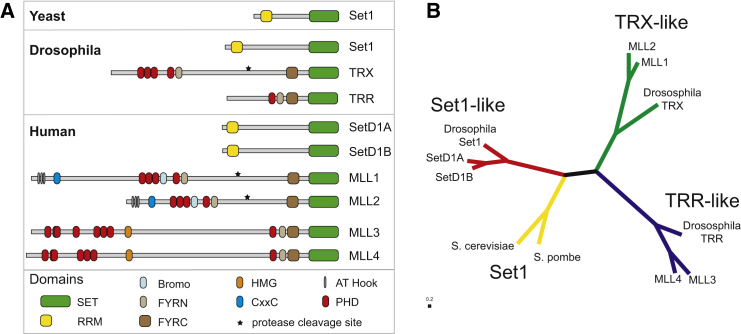
The KMT2 Family of Histone H3K4 Methyltransferases (A) The domain architecture of the KMT2 proteins found in yeast, *Drosophila*, and humans. (B) Phylogenetic analysis based on the sequence of only the SET domains of KMT2 proteins. The proteins segregate into the same groups in both sets of proteins. See also the extended sequence alignment in Figure S1.

Histone methyltransferases and demethylases form a major part of the highly dynamic chromatin modification system that enables epigenetic regulation. Methylation of the lysine-4 residue on histone H3 (H3K4) facilitates the recruitment of transcriptional complexes and correlates well with active gene transcription (Bannister and Kouzarides, 2011; Ruthenburg et al., 2007; Smith et al., 2011). These marks play an essential role in organizing gene expression, and as such their placement must be tightly regulated. With increased complexity, elaborate regulatory mechanisms have evolved in eukaryotic cells to control chromatin modifications (reviewed in Kusch, 2012; Shilatifard, 2012). Consequently, in yeast, a single methyltransferase complex, Set1, is responsible for all H3K4 histone methylation, whereas in humans, the homologous MLL (or KMT2) family has expanded to six members (Kusch, 2012; Miller et al., 2001; Roguev et al., 2001; Schlichter and Cairns, 2005). The different family members can be distinguished by the pattern of their targeting domains (such as PHD fingers, BROMO domains, or RRM domains) (Figure 1A). These proteins, MLL1 and 2, MLL3 and 4, and SetD1A and B (or KMT2a to KMT2f), have most likely arisen from duplications of ancestral genes that encoded proteins similar to the *Drosophila* Set1, TRX, and TRR (Morgan and Shilatifard, 2013).

The individual roles of each MLL protein are not fully understood, but recent studies have revealed insights into their separate functions (Denissov et al., 2014; Hu et al., 2013; Lee et al., 2013). The emerging picture is that, whereas the SetD1A and SetD1B proteins may be responsible for global H3K4 methylation, the TRX-like and TRR-like proteins have more specialized roles (Bledau et al., 2014; Hallson et al., 2012). For example, the MLL1 and MLL2 proteins are implicated in the regulation of only a small number of *Hox* genes in early development (Denissov et al., 2014). The TRR-like MLL3 and MLL4 methyltransferases are implicated in the regulation of a slightly broader subset of genes. For example, promoters shown to bind MLL4 include those of p53, cyclic AMP signaling genes, and retinoic acid receptors (Guo et al., 2012). Disruption of different MLL proteins is associated with different disease pathways; notably, it has long been known that chromosomal translocations that disrupt MLL1 can contribute to aggressive leukemias (Dou and Hess, 2008). However, mutations in MLL4 are linked to the congenital abnormality Kabuki syndrome (Micale et al., 2014).

In humans, MLL proteins are relatively large in size. The smallest SetD1a has 1,707 amino acids and the largest MLL4 has 5,537. However, the conserved H3K4-specific catalytic SET domain is a small component at the C terminus, comprising only 150 amino acids. A defining feature of the MLL family, conserved through evolution, is that their SET domain must associate with a multiprotein complex for full catalytic activity (Dehe et al., 2006; Dou et al., 2006; Roguev et al., 2001; Yokoyama et al., 2004). Analyses indicate that additional components may associate with different MLL proteins, but that all bind to a conserved core complex (Hu et al., 2013; Van Nuland et al., 2013). This core multiprotein complex comprises four subunits, WDR5, RbBP5, Ash2L, and Dpy-30, and is commonly referred to as the WRAD complex. Mutation or downregulated expression of WRAD proteins leads to a loss of the methyltransferase activity associated with MLL proteins, thus implying the MLL SET domain must associate with WRAD for activation (Dehe et al., 2006; Dou et al., 2006; Roguev et al., 2001).

The molecular basis of WRAD-mediated stimulation of MLL methyltransferase activity has been the subject of a number of studies, reviewed in Cosgrove and Patel (2010) and Ernst and Vakoc (2012). All MLLs have a conserved arginine-containing motif N-terminal to the SET domain, termed the WIN motif, which binds WDR5 (Patel et al., 2008; Zhang et al., 2012). This appears to form a hub that facilitates the recruitment of the other components of the complex (Avdic et al., 2011; Couture and Skiniotis, 2013; Odho et al., 2010; Tremblay et al., 2014). Recent evidence indicates that the assembly process may be regulated by posttranslational modification, for example through phosphorylation of RbBP5 (Zhang et al., 2015). The crystal structure of the isolated MLL1 SET domain revealed an open conformation, which was suboptimal for methyl transfer to the target lysine (Southall et al., 2009). This led to the hypothesis that the interaction with WRAD components induced a more optimal SET domain conformation, thus stimulating activity. However, the detailed mechanism of stimulation of methyltransferase activity by WRAD is not yet fully established. In in vitro studies, methylated histone product could be detected following incubation with WRAD complex reconstituted with an inactivated SET domain (Patel et al., 2011; Shinsky et al., 2014). This led to the hypothesis that assembly of the MLL SET domain with WRAD generates a cryptic *S*-adenosylmethionine (SAM) binding site, which potentially participates in di- and trimethylation events. The molecular details of the pairwise interactions between the MLL SET domain and different WRAD subunits are beginning to emerge (Cao et al., 2014). An electron microscopy reconstruction has revealed the overall shape of the homologous yeast COMPASS complex, which provides a clear indication of the relative position of the subunits (Takahashi et al., 2011). The yeast and mammalian complexes are conserved, and MLL is expected to retain a similar overall conformation.

Variation in the relative in vitro methyltransferase activity of different MLL family members has been reported (Cao et al., 2014; Shinsky et al., 2015; Zhang et al., 2012). Consistently MLL1 exhibits the weakest activity in the absence of WRAD complex, and in a recent study MLL3 intrinsic activity was substantially higher than that of the other MLLs (Zhang et al., 2012). Differences have also emerged in how the methyltransferase activity of individual MLL proteins may be controlled. For example, in their investigation of the effect of H2B ubiquitination on MLL complex activity, Dou and colleagues found that MLL1 and SetD1A activity was positively regulated by ubiquitination, whereas for MLL3 there was no discernible effect (Wu et al., 2013b). In a study of compounds that inhibit H3K4 methylation, MLL1 was found to be more sensitive than the other members of the family to compounds that target the central binding site of the WDR5 propeller (Cao et al., 2014).

Using only the sequence of the C-terminal SET domains, the MLL family segregates into three distinct groups (Figure 1B). Intriguingly these subgroups reproduce those created using the domain architecture of the full proteins. Together with differences in expression, this implies that the different MLL proteins are uniquely targeted. As the sequence analysis indicates that their SET domains have specialized, we were interested in whether SET domains from different subgroups had evolved distinct intrinsic enzymatic properties. MLL1 (KMT2A located on human chromosome 11) and MLL4 (KMT2D located on human chromosome 12) were selected as representatives of the TRX-like and TRR-like groups, respectively. Here, we present the characterization of the MLL4 SET domain, and show that it has catalytic properties distinct from those of MLL1. To explain how these differences arise from small changes in primary sequence, we have determined the crystal structure of the MLL4 SET domain (using a construct inactivated by its C-terminal tag) and compared it with the previously published MLL1 structure (Southall et al., 2009). From this analysis, in addition to confirming a role for the orientation of the SET-I region in activation, we have identified a previously unknown function for the postSET loop in the MLL activation mechanism.

## Results

### MLL1 and MLL4 Have Different Catalytic Properties

**Figure 2:**
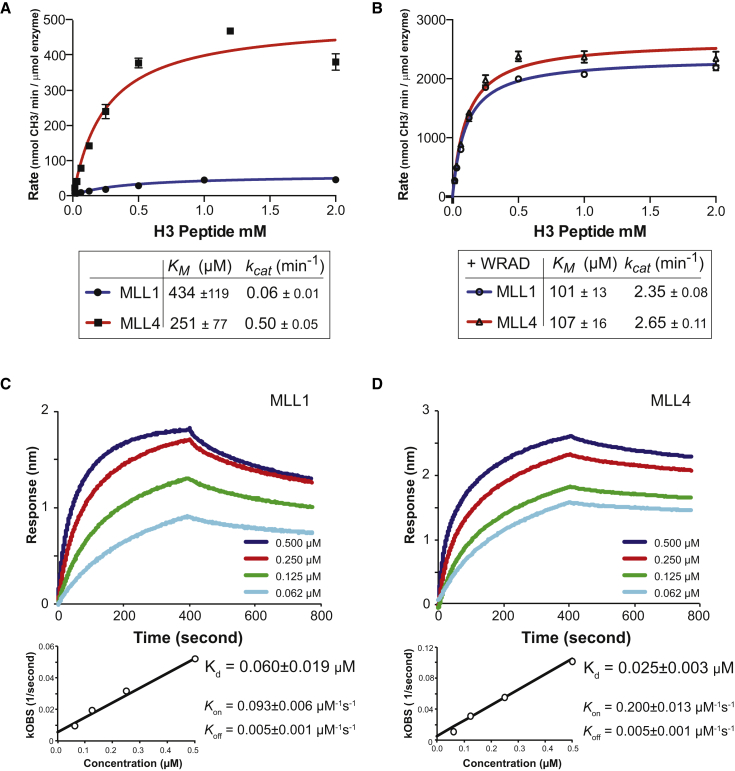
Activity and WRAD Binding of the MLL1 and MLL4 SET Domains Details of the selection of assay conditions are provided in Figure S2. (A) Methyltransferase activity of MLL1 and MLL4 SET domain constructs with saturating SAM and increasing H3 peptide substrate. MLL4 shows much higher methyltransferase activity than MLL1 with respect to peptide substrate. (B) Methyltransferase activity of MLL1 and MLL4 SET domain constructs plus WRAD complex with saturating SAM and increasing H3 peptide substrate. Both MLL1 and MLL4 show significantly higher activity in the presence of WRAD complex. Quantification of methyl transfer was calculated from liquid scintillation measurement of recovered peptide, assuming 1 dpm is equivalent to 5.68^−9^ nM CH_3_. Error bars represent the standard deviation of triplicate measurements. (C and D) Binding kinetics of immobilized MLL1 SET domain (C) and MLL4 domain (D) to WRAD complex. Both MLL1 and MLL4 SET domain constructs bind tightly to the WRAD complex.

Although well conserved, the SET domains of MLL1 and MLL4 have a sequence identity of 47% and a similarity of 68%. We looked for a correlation between their segregation into different subgroups and their catalytic properties. Equivalent recombinant constructs of these two proteins were prepared containing the SET domain (starting from the conserved WDR5 binding “WIN” motif to the C terminus). First, we determined whether, like MLL1, the MLL4 SET construct had minimal catalytic activity in the absence of the core complex. A methyltransferase assay was performed with both enzymes under identical conditions, with saturating SAM and a range of H3 peptide substrate concentrations (Figure 2A). In the absence of the WRAD complex, the MLL1 SET domain exhibited weak activity with a *K*_M_ (H3 peptide) of 430 μM and a turnover rate (*k*_cat_) of 0.06 min^−1^. However, the equivalent MLL4 SET domain construct had approximately ten times more activity (*k*_cat_ 0.50 min^−1^) (Figure 2A). The MLL4 *K*_M_ (peptide) was estimated to be 251 μM, and thus similar to that of MLL1, showing that the higher intrinsic activity of MLL4 is not likely to arise from differences in substrate binding affinity.

The need to associate with the WRAD complex for full activation is a well-established principle for MLL SET domains (Couture and Skiniotis, 2013; Ernst and Vakoc, 2012; Shilatifard, 2012). To compare the effects of WRAD-mediated stimulation of MLL4 and MLL1 proteins, we coexpressed the WRAD complex (WDR5, RbBP5, Ash2L, and Dpy-30) in insect cells, and purified it using a Strep affinity tag on the RbBP5 subunit. Methyltransferase assays with the MLL1 and MLL4 WIN constructs were then repeated in the presence of equimolar WRAD (Figure 2B). The methyltransferase activity of both MLL1 and MLL4 increased when complexed with WRAD. However, in identical experimental conditions for MLL1 we observed a 40-fold increase in *k*_cat_ over the intrinsic activity, while *k*_cat_ of MLL4 increased only 4-fold. To determine whether the higher stimulation of MLL1 by WRAD was a consequence of a higher affinity for the complex, we performed protein-protein interaction experiments using biolayer interferometry. MLL1 and MLL4 constructs containing the WDR5 binding WIN motif were immobilized on a sensor chip, and the binding of WRAD was measured (Figures 2C and 2D). WRAD complex bound tightly to both enzymes; the estimated *K*_d_ for MLL1-WRAD and MLL4-WRAD was 60 nM and 25 nM, respectively. Thus at the WRAD and SET domain concentrations used in assays, we estimate that approximately 93% MLL1 and 95% MLL4 should be present as complex. For both MLL1 and MLL4-WRAD complexes the observed enzymatic activity was similar, with *K*_M_ (100 μM) and *k*_cat_ (2.35 min^−1^ and 2.65 min^−1^), respectively (Figure 2B). Importantly, although MLL4 has significantly higher intrinsic activity, both enzymes were only fully activated in complex with WRAD.

### The Structure of the MLL4 SET Domain

**Figure 3:**
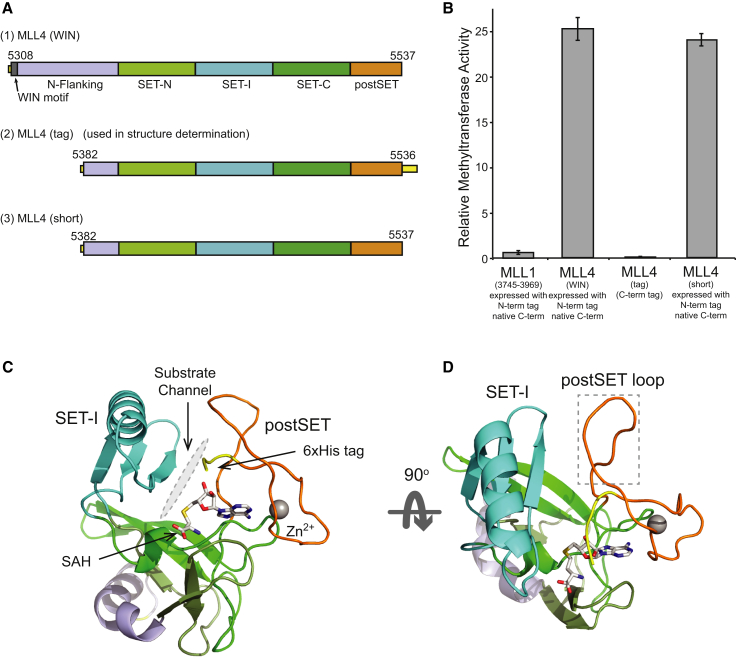
Structure of the MLL4 SET Domain (A) Schematic representation of MLL4 constructs used in this study, indicating the subdomains of the MLL4 SET domain and providing a key for colors used in (C) and (D). (B) Methyltransferase activity of SET domain constructs used in this study. Activities are presented relative to the MLL1 (WIN) construct. Further details of the constructs used and their relative activity are presented in Figure S3. Error bars represent the standard deviation of triplicate measurements. (C) Cartoon representation of MLL4 in two orthogonal orientations. SET domain regions are colored as indicated in the key. The protein is shown in cartoon representation with the C-terminal 6xHis tag shown in yellow. The cofactor product, *S*-adenosyl homocysteine, is shown in stick representation, and the single coordinated Zn^2+^ ion as a sphere. The substrate binding channel, inferred from the structure of MLL1, is indicated in gray. (D) Orthogonal view of the MLL4 SET domain indicating the relative positions of the SET-I region and postSET loop. The box indicates the position of the postSET loop.

To address the molecular basis of the higher intrinsic activity of the MLL4 SET domain, we determined its crystal structure. The MLL4 (WIN) construct used in kinetic studies did not yield crystals despite exhaustive attempts, so a combinatorial domain hunting procedure was used to identify a construct suitable for crystallography (Reich et al., 2006). An expression library of MLL4 gene fragments was generated and screened for soluble expression in *Escherichia coli*. From an initial library diversity of 157,000 clones, 25,000 colonies were screened and 18 unique, well-expressed, and soluble constructs were identified that covered both the SET and postSET domains. The final construct used for structural determination, MLL4(tag), encompassed residues 5,384 to 5,536, followed by a C-terminal 6xHis tag, and crystallized with *S*-adenosylhomocysteine (SAH) cofactor product (Figures 3A and S3). The crystals diffracted to a resolution of 2.2 Å and belonged to the space group P2_1_. The structure was determined by molecular replacement using the central core of the MLL1 SET domain structure as the search model (Southall et al., 2009), and the relevant crystallographic statistics are presented in Table 1.

The catalytic domain of MLL4(tag) adopts the canonical β-fold structure observed for other SET domains, and closely resembles that of MLL1 (Figures 3C and 3D) (Dillon et al., 2005; Southall et al., 2009; Xiao et al., 2003b). The N-flanking region of MLL4 forms an extended helical structure, and is markedly different from the extended loop observed for MLL1. Conserved features include the cofactor binding site, which is located in a surface pocket created by the intersection of the SET-N, SET-C, and postSET domains. The position of the substrate binding channel is located between the SET-I and postSET domains, and is indicated in Figure 3C. The C-terminal tag immediately follows the conserved Cys_4_Zn cluster and enters the substrate binding channel before turning and passing over the cofactor. The tag was required for crystallization but resulted in a catalytically dead construct (Figure 3B), and its impact on the MLL4 SET domain structure and activity is discussed in detail in Figure S3. An equivalent MLL4 construct prepared with an N-terminal tag, like the MLL4(WIN) construct, had high intrinsic activity (Figure 3B), indicating that the determinants of this intrinsic activity are contained within this region of the SET domain. The lack of methyltransferase activity observed with the C-terminally tagged construct can be attributed to partial blocking of the substrate binding channel and interactions with the cofactor.

### Comparison of the MLL1 and MLL4 SET Domain Structures

**Figure 4:**
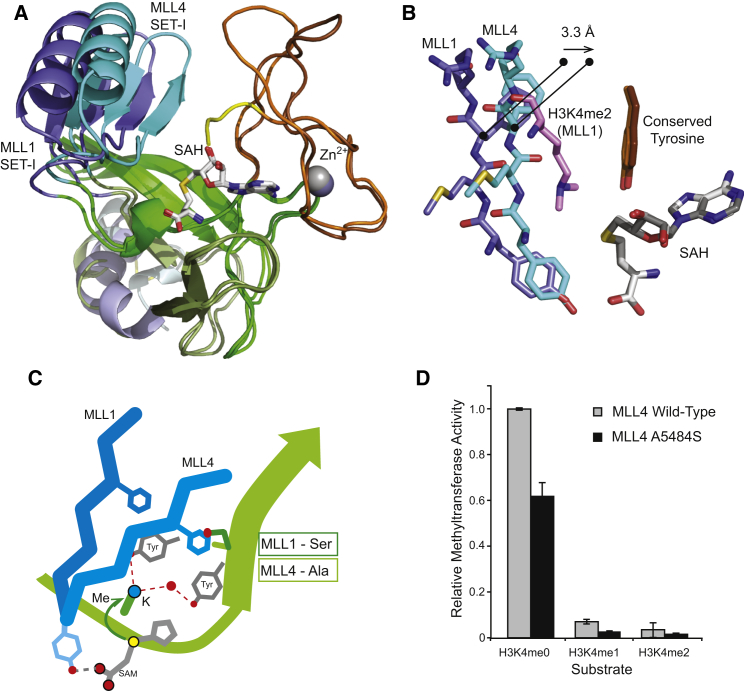
Comparison of the MLL1 and MLL4 SET Domain Structures (A) Superposition of the MLL1 (darker colors) and MLL4 SET domains in carton representation. Superposition performed in Coot on the cofactor molecule. (B) Detail of the position of the channel tetrapeptide segments of MLL1 and MLL4, showing how the MLL4 construct better constrains the target lysine substrate due to a shift of approximately 3.3 Å toward the Tyr5510 residue (MLL4 numbering). (C) Schematic representation showing the relationship of the channel tetrapeptide with the cofactor and SET-C strand. The Ala residue on the SET-C strand forms a closer interaction with the tetrapeptide Phe in MLL4. (D) Mutation of the MLL4 Ala5484 residue to Ser reduces the intrinsic methyltransferase activity of the construct relative to the wild-type. Further structural details are presented in Figure S4. Error bars represent the standard deviation of triplicate measurements.

Previously, we observed that in the MLL1 SET domain structure, the SET-I region lay further from the active site than in the structures of other SET domain proteins (Couture et al., 2005; Southall et al., 2009; Xiao et al., 2003a; Zhang et al., 2002). We argued that this contributed to the weak intrinsic activity observed for MLL1, as the target lysine is not held in the optimal position for methyl transfer. To compare the MLL4(tag) structure with MLL1, the two structures were aligned using their SAH cofactors as the reference (Figure 4A). The SET-N, SET-C, and postSET Zn^2+^ ion binding regions all superpose closely, with a root-mean-square deviation of only 0.8 Å for Cα atoms over this region. However, two elements of the SET domain, the SET-I region and the postSET loop, adopt different positions in the two structures.

In MLL4(tag) the SET-I region, which retains the conserved fold observed in MLL1 and other SET domain structures, is in a position that is more consistent with the “closed” conformation proposed for an active SET domain (Southall et al., 2009). The consequence of this position for the MLL4 SET-I region is that the four-residue motif, termed the channel tetrapeptide (I-Y-M-F in MLL4), is 3.3 Å closer to the conserved active-site tyrosine that flanks the target lysine side chain (Tyr5512 residue in MLL4) relative to MLL1 (Figure 4B). The target lysine side chain would therefore have less freedom of movement in MLL4, which would promote the S_N_2 nucleophilic methyl transfer reaction and increased catalytic efficiency. The catalytic efficiency (*k*_cat_/*K*_M_) for MLL4 was 2.0 × 10^−3^, compared with 1.0 × 10^−5^ for MLL1. When complexed with WRAD, the *k*_cat_/*K*_M_ of both enzymes increased to approximately 2.5 × 10^−2^. The position of the SET-I region relative to the postSET Tyr observed in the MLL4(tag) structure could therefore represent an intermediate state, between the position in the deactivated conformation observed for MLL1 and that when fully activated by WRAD. We suggest that WRAD association with the MLL4 SET domain stabilizes a fully activated conformation.

**Figure 5:**
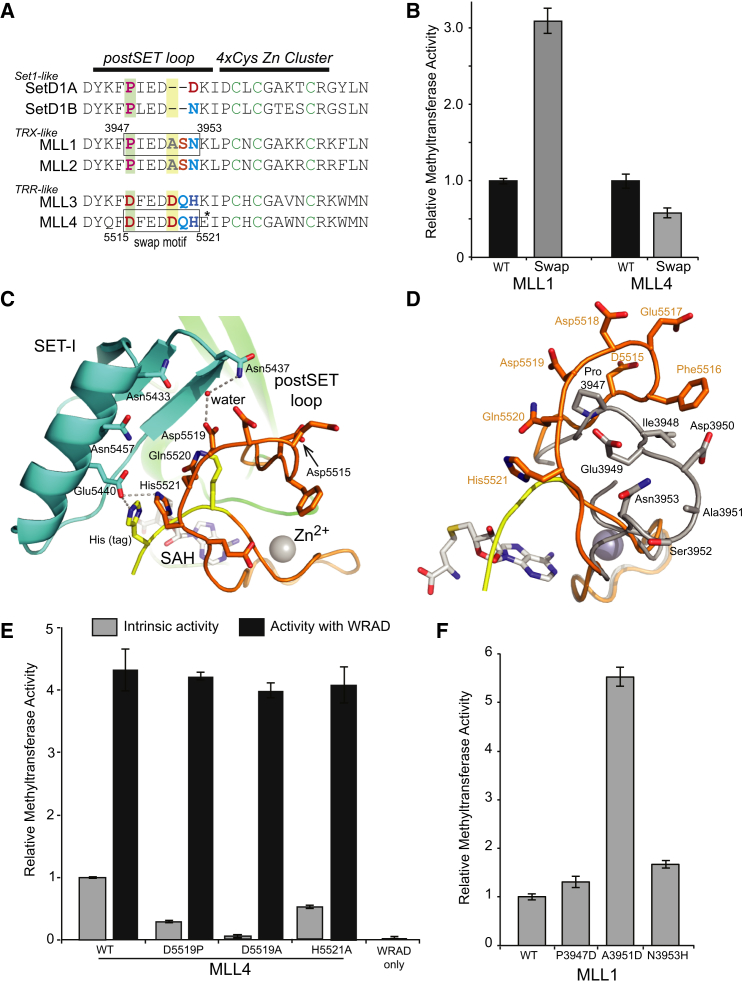
Role of the PostSET Loop in Enzyme Activation (A) Sequence of the postSET regions of the MLL methyltransferases. Position 5522 in MLL4, indicated by an asterisk, is Lys in some database entries. The boxed sequence indicates the motif swapped between MLL1 and MLL4. (B) The effect of swapping the postSET loop regions of MLL1 and MLL4 on methyltransferase activity, relative to respective wild-type (WT). Error bars represent the standard deviation of triplicate measurements. (C) The MLL4 postSET loop (orange) makes potential interactions with the SET-I region (cyan). The C-terminal tag is indicated in yellow. (D) Superposition of the MLL1 and MLL4 postSET regions, showing that the MLL1 postSET loop (gray) is located further from the SET-I region than that of MLL4 (orange). (E) Site-specific mutations to the MLL4 postSET loop have a detrimental effect on their activity relative to the WT construct. However, activity is recovered in the presence of WRAD complex. (F) The effect of reverse mutations in MLL1 on activity.

Can sequence features be identified that account for the relative positions of the SET-I region in MLL1 and the MLL4(tag) structures? The active site in SET domain proteins is enclosed by three elements: the SAM cofactor, the SET-C region, and the channel tetrapeptide segment (Figure 5C). At its N terminus the tetrapeptide is connected to the cofactor by a hydrogen bond with the conserved tyrosine (see Figures 4C and S4). A bulky side chain, often a phenylalanine, for example in Ezh2, Suv39, or Dim5, then makes a stabilizing interaction with a hydrophobic residue on the first β strand of the SET-C region. As Figure 4C shows, in MLL4 the phenylalanine packs against an alanine on the SET-C strand, but in MLL1 there is a serine in the equivalent position on SET-C, so that the equivalent hydrophobic interaction cannot be made. Interestingly, mutation of the MLL4 SET-C alanine (Ala5484) to a serine results in an approximately 2-fold reduction in MLL4 methyltransferase activity (Figure 4D). It is likely that several sequence features combine to promote the partially activated conformation and higher intrinsic activity we observe for MLL4. It should also be noted that the side chain of the first histidine of the MLL4 C-terminal tag makes a hydrogen bond to the side chain of the SET-I residue Glu5440. Although it is not possible to rule out that this may influence the position of SET-I, this seems unlikely given the inherent flexibility of the tag, and this issue is discussed in greater detail in Figure S3.

### Role of the postSET Loop in Activation

A short loop region, termed the postSET loop (Figures 3C and 3D), links the well-conserved postSET Cys_4_Zn cluster and the SET-C region. The sequence of this loop region, which comprises residues 5,515–5,521 in MLL4, varies significantly among the different MLL subgroups (Figure 5A). Although the loop is the same length in MLL1 (TRX) and MLL4 (TRR), the difference in the sequence of the TRX-like and TRR-like groups is conspicuous given the otherwise high degree of conservation across the domains. The sequence within the subgroups, for example between MLL3 and MLL4, is conserved. We were interested in whether these differences in the postSET loop contributed to the higher intrinsic activity of MLL4. We prepared chimera constructs in which the MLL1 and MLL4 postSET loop motif was “swapped.” The intrinsic methyltransferase activity of the “swap” constructs was compared with their respective wild-type parent SET domains (Figure 5B). Replacing the MLL1 sequence with that of MLL4 led to a 3-fold increase in the methyltransferase activity, while the reverse substitution reduced MLL4 activity by about 2-fold. The domain swap data are consistent with the sequence of the postSET loop region of the TRR-like proteins contributing to their intrinsic enzyme activity.

Previous analyses of SET domain structures have indicated that the postSET loop is a flexible feature linking the SET-C to the Cys_4_Zn cluster (Dillon et al., 2005). In crystal structures of SET domains that have a Cys_4_Zn cluster in their postSET region, such as Dim5, Suv39H2, and EzH2, the postSET loop was disordered, and therefore was not included in the final atomic model (Wu et al., 2010, 2013a; Zhang et al., 2002). Indeed, in our previously reported structural analysis of the MLL1 SET domain, although we were able to build the postSET loop in the crystal structure of the ternary complex with peptide and SAH, it was not possible for the equivalent binary complex without peptide (Southall et al., 2009). In the current structure of the MLL4 SET domain, the postSET loop has B factors notably higher than those in other regions. This again highlights the flexibility of this region. Nevertheless, electron density maps, although weak, were good enough to build in this segment. Inspection of the MLL4 structure reveals that several side chains of residues in the postSET loop are oriented toward the SET-I region, and some are capable of making hydrogen bonds with it (Figure 5C). Although due to the inherent flexibility of the loop these interactions may be transient, they may contribute to MLL4 intrinsic activity. In the MLL1 ternary complex structure the postSET loop adopts a conformation that is much more distant from the SET-I region, and there are no implied interactions between these two regions (Figure 5D). Our interpretation is therefore that while the MLL postSET loop is inherently flexible, the biochemical data support the idea that sufficient interactions can be made with SET-I that contribute to activity.

We were interested in the contributions of individual postSET loop residues to activity. The conformation of the MLL1 and MLL4 postSET loops start to diverge at the MLL4 Asp5515 residue, which is equivalent to Pro3947 in MLL1 (Figures 5A and 5D). The MLL4 D5515P mutant had reduced methyltransferase activity compared with wild-type (Figure 5E), although the reverse mutation in MLL1, P3947D, led to only a modest increase (Figure 5F). Two MLL4 postSET residues are close enough to interact with SET-I residues Asp5519 and His5521 (Figure 5C). Asp5519 is centrally located in the loop and could make a water-mediated hydrogen bond with Asn5437 on SET-I. In MLL4 the D5519A mutation effectively abolished the intrinsic methyltransferase activity (Figure 5E). In MLL1 the equivalent residue is Ala3951, and a MLL1 (A3951D) mutant had approximately 6-fold higher methyltransferase activity compared with wild-type (Figure 5F). The His5521 residue may make a relatively weak hydrogen bond to SET-I residue Glu5440, although this interaction may be influenced by the second His tag residue, which also interacts with Glu5440. The MLL4 (H5521A) mutation reduced intrinsic MLL4 activity by about 2-fold, and the reverse mutation in MLL1 (N3953H) led to a modest increase in activity. The MLL4 Asp5519 had the most significant effect on intrinsic activity but, interestingly, when the MLL4 (D5519A) mutant was reconstituted with WRAD complex, in common with the other postSET loop mutants the activity was restored to near wild-type levels (Figure 5E). A role for the postSET loop has been previously proposed in substrate/cofactor product release (Dillon et al., 2005; Zhang et al., 2003). We propose that the inherent flexibility of the postSET loop region means that in addition it makes a transient interaction with SET-I, contributing to the stabilization of the active conformation in MLL4. The addition of WRAD complex then further stabilizes the active conformation.

### MLL Product Specificity

**Figure 6:**
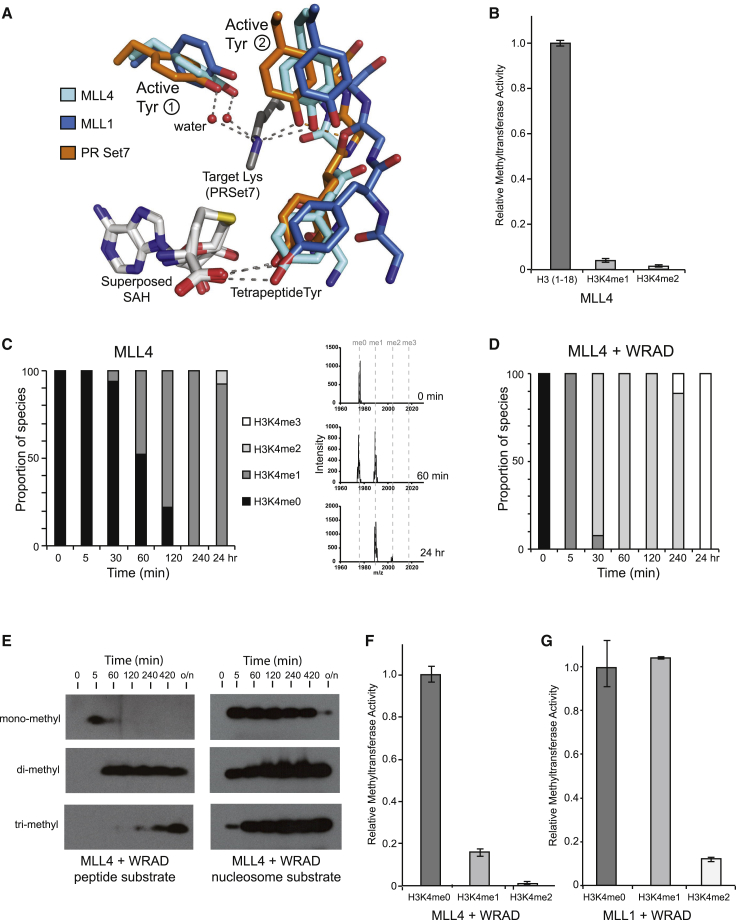
Product Specificity of the MLL4 SET Domain (A) Superposition of the active sites of MLL1 (blue), MLL4 (cyan), and PR-Set7 (orange) indicating the positions of the channel tetrapeptide residues and Phe/Tyr switch residues. Conserved hydrogen bonds are indicated by gray dots, while the tetrapeptide main chain to active-site tyrosine hydrogen bond is shown by orange dots. This interaction is affected by the position of the channel tetrapeptide in MLL1 and MLL4. Here the main-chain carbonyl of the next tetrapeptide residue is within hydrogen bonding distance of the active-site tyrosine hydroxyl (3.5 Å in both structures), probably contributing to a displacement of this residue compared with the position observed in PR-Set7. (B) MLL4 shows intrinsic monomethylase specificity. Error bars represent the standard deviation of triplicate measurements. (C) Analysis of the reaction products at specified time points following incubation of MLL4 with unmodified peptide substrate. Different methyl species are indicated by the shading in the adjacent key. Representative MALDI-TOF spectra are shown to the right, and the full range of spectra is presented in Figure S5. (D) Analysis of the generation of H3K4 methyl species using MLL4/WRAD complex, shaded as in (C). (E) Gel-based methyltransferase assay with MLL4/WRAD reconstituted complex using peptide and nucleosome substrate. Reactions were stopped at the indicated times by boiling in sample buffer. The resulting blot was probed with antibodies specific to mono-, di-, or trimethylated H3K4 (Abcam). (F and G) Addition of WRAD to the MLL4 or MLL1 SET domain increases the overall methyltransferase activity, but not the pattern of substrate preference. Note that these assays were performed at an enzyme concentration of 25 μM.

The phenylalanine/tyrosine switch hypothesis is a well-established rule of thumb for predicting the product specificity of SET domain proteins based on the sequence of their active site (Couture et al., 2008; Xiao et al., 2003b; Zhang et al., 2003). Essentially, if the two residues in the active site that constrain the target lysine Nε are both tyrosines, the SET domain is restricted to monomethylation. However, SET domains with a phenylalanine in the position of one of these “active-site tyrosines” also have the capacity for di- and trimethylation activity. Many studies have reported mono-, di-, and trimethylation of H3K4 for MLL methyltransferases (reviewed in Kusch, 2012). However, across the MLL family both the active-site residues are conserved tyrosines (Figure S1). Superposing the MLL4(tag) structure with PR-Set7 confirms that these residues occupy the same positions in the active site as this well-established monomethylase (Figure 6A). Using H3 substrate peptides that were either unmodified, monomethylated, or dimethylated at the K4 position, we measured the intrinsic product specificity of the MLL4 SET domain construct (Figure 6B). We found that it had a significant preference for the unmodified peptide substrate and, in line with the sequence and structural prediction, indicates that MLL4 has intrinsic monomethylase activity.

In common with the other MLL proteins, MLL4 has been associated with mono-, di-, and trimethylation of H3K4 in cellular studies (Issaeva et al., 2007; Lee et al., 2013). We used MALDI-TOF mass spectrometry to investigate the methylated state of histone peptide following incubation with MLL4 (Guitot et al., 2014). An unmodified histone peptide was incubated with an equimolar concentration of enzyme, and the reaction products were analyzed at specified time points (Figure 6C). After 60 min of incubation an approximately equal amount of unmodified and monomethylated species was detected. Following overnight incubation, dimethyl species was also observed. However, with the addition of WRAD the peptide was rapidly converted to the monomethylated species (Figure 6D). After 30 min of incubation the dimethylated species dominated, and a trimethylated species was detected following a longer incubation of 4 hr. After overnight incubation the trimethylated species was the only product detected. Thus, the MLL4 SET domain permits the generation of multiple methyl species and, in complex with WRAD, the reaction proceeds more rapidly. Interestingly, the Couture group has recently reported that the second TRR-like subgroup member, MLL3, can also introduce multiple methyl groups to an unmodified H3 substrate, in this case when in complex with a Ash2L/RbBP5 heterodimer (Zhang et al., 2015). In their assays, in the absence of the Ash2L/RbBP5 heterodimer, only monomethyl product was detected using the MLL3 SET domain.

Conversion of the unmodified peptide substrate to the trimethyl species by MLL4/WRAD was also observed in a gel-based assay format using a set of methyl-specific antibodies to analyze the product (Figure 6E). In this experiment the substrate peptide was in 20-fold excess of the enzyme complex. Using this assay format, we were also able to follow the generation of different methyl species using recombinant mononucleosomes as a more physiologically representative substrate. Again the signal for monomethyl H3K4 peaked after 5 min, but had disappeared after overnight incubation. Meanwhile the trimethyl product gradually accumulated over the course of the reaction.

Can the MLL1 and MLL4(tag) structures explain how MLL proteins are able to catalyze multiple methylation steps? We have previously reported that an MLL1 SET domain construct was able to methylate both unmodified and monomethylated H3K4 peptide, implying that it had both intrinsic mono- and dimethylase activity (Southall et al., 2009). WRAD association stimulates the overall methyltransferase activity of both MLL1 and MLL4 SET domains (Figure 2B). This increase in activity was also observed using premodified H3K4me1 and H3K4me2 substrates, but the pattern of substrate preference remained broadly in line with that observed for the SET domain alone (Figures 6F and 6G). Thus for MLL4 the active site is best suited to monomethylation, and this is the most efficient reaction it catalyzes. Importantly, however, the higher overall activity in association with WRAD means that the di- and trimethylation activity, although still lower than for monomethylation, becomes more easily detected.

It is striking that in these assays MLL1 is better than MLL4 at catalyzing multiple methylations (Southall et al., 2009; Figure 6G), even though the key residues defining the active site are conserved and occupy similar positions to those observed in MLL4 and PR-Set7 (Figure 6A). However, the more open position of the MLL1 channel tetrapeptide residues, discussed above, also has implications for the position of the active-site residues. In PR-Set7 the tetrapeptide tyrosine residue, in addition to forming a hydrogen bond with its side chain and the SAM cofactor, makes a good hydrogen bond (H-bond geometry distance 2.7 Å) between its main-chain carbonyl and the hydroxyl of the second active-site tyrosine, Tyr(2) (Figure 6A), thus helping to constrain the position of this residue. In the MLL proteins the orientation of the tetrapeptide main chain is different, and the distance between the equivalent hydrogen bond groups increases (3.5 Å in the MLL4 structure and 3.8 Å in MLL1). As a result, the active site Tyr(2) to target lysine Nε distance is greater in the MLL4(tag) structure than in PR-Set7, and even greater in MLL1. There is now sufficient space around the target lysine Nε to accommodate multiple methyl groups, and indeed a dimethyl lysine substrate product was cocrystallized in the MLL1 structure (Southall et al., 2009). Thus, we propose that the mobility of the tetrapeptide element in MLL SET domains could contribute to their partial non-compliance with the phenylalanine/tyrosine switch rule.

## Discussion

The high demand for regulation of histone H3K4 methylation at chromatin sites has led to the expansion of the MLL family to six members in higher eukaryotes. In addition, the MLL methyltransferase domains must assemble into a multiprotein complex for full catalytic activity. The MLL SET domains segregate into three classes based only on the sequence of this 150-amino-acid motif, and these are the same groupings that arise through classification based on their targeting domains. This suggests that the different MLL SET domains may have evolved discrete properties linked to their different targeting features. The residues that define cofactor and substrate recognition are absolutely conserved, indicating that methylation of H3K4 is their core function. Here we have shown that the representatives of the TRX-like and TRR-like subgroups, MLL1 and MLL4, respectively, differ in their intrinsic catalytic properties. Specifically, MLL4 has 10-fold higher methyltransferase activity than MLL1, and had a stronger preference for monomethylase product specificity. Upon association with the WRAD complex the observed activity of both proteins increased, with the MLL1/WRAD and MLL4/WRAD complexes exhibiting similar kinetic behavior. From this we infer that when associated with WRAD, both proteins adopt a similar optimal conformation when fully activated. This implies that the structure of the isolated MLL4 SET domain, with respect to the positions of the SET-I region and postSET loop, may represent an intermediate state, which is partially stabilized by features unique to MLL4.

Superposing the MLL1 and MLL4 SET domain structures revealed a significant difference in the position of the SET-I region. The position observed in the current MLL4 structure is more consistent with that of SET domains of structures that do not require additional components for activation, for example, PR-Set7, Set7/9, or Dim-5 (Couture et al., 2005; Xiao et al., 2003a; Zhang et al., 2002). Although they had significantly different intrinsic activity, we found that in complex with WRAD both enzymes reached a similar overall level of activity. In this model for activation, the crystal structures of the MLL1 and MLL4 isolated SET domains would represent snapshots of different stages of the activation mechanism. We speculate that WRAD binding may induce a further movement in SET-I, resulting in an even more closed active site. MLL1 was more permissive than MLL4 with regard to product specificity, even when associated with WRAD complex. We propose that the more open conformation observed for the isolated MLL1 SET domain structure implies greater flexibility, and that this may contribute to the observed product specificity. Following this line of reasoning would require that even when in complex with WRAD, MLL1 SET-I retains more flexibility than MLL4. Flexibility of MLL SET domain regions may be linked to cofactor turnover, and it may therefore be significant that there is contact between the conserved channel tetrapeptide tyrosine and the cofactor.

The flexibility of the postSET loop has previously been linked to cofactor turnover (Dillon et al., 2005). It is interesting that in the MLL4(tag) structure we have captured MLL4 in a conformation that suggests this region may also have an additional role in activation. Although this structural analysis suggests that interactions with SET-I may only be transitory, deactivating mutations in MLL4, and the reverse mutations that activate MLL1, support a role for interactions made by these residues in activation. Potential interactions the postSET loop may make with substrate have not been tested in our current analysis, but may also contribute to intrinsic activity. It is significant that WRAD association compensates for the postSET mutations in MLL4, such as D5519A, that were detrimental to intrinsic activity. One hypothesis proposed to explain WRAD activation of MLLs is that WRAD subunits form a surface with MLL that creates a secondary active site (Shinsky et al., 2014). Our new data do not contradict this model. However, we favor a model in which the WRAD association with the MLL SET domain stabilizes a fully activated conformation, consistent with our previous observations that the individual members of WRAD act cooperatively to enhance MLL1 activity (Odho et al., 2010; Southall et al., 2009). The features in the postSET region of MLL4 therefore stabilize an intermediate conformation. A fully formed MLL-WRAD complex is the optimally active configuration for all MLL methyltransferases. However, our data, together with those showing intrinsic activation of MLL3 (Zhang et al., 2012), suggest that there may be a hitherto unidentified function for TRR-like proteins utilizing monomethylation of H3K4 independent of the complex.

## Experimental Procedures

### Sequence Analysis

Multiple sequence alignments were performed using the T-Coffee server (tcoffee.crg.cat). For phylogenetic analysis, the sequence from the SET-N region to the C terminus was used, the tree was produced in T-Coffee, and the tree diagram was produced using the Phylodendron phylogenetic tree printer (Indiana University). To generate pairwise identity/similarity statistics, the EMBOSS-needle algorithm for pairwise sequence alignment (McWilliam et al., 2013) was used.

### Protein Production

Human MLL4(5308–5537), MLL4(5382–5537), and MLL1(3745–3969) SET domain constructs were expressed as glutathione *S*-transferase (GST)-fusion proteins. The MLL4(5382–5536), used for crystallization, was expressed with a C-terminal 6xHis tag. All constructs were expressed in *E. coli* BL21 cells (Agilent). GST-fusion proteins were isolated from clarified lysates using glutathione Sepharose affinity resin (GE Healthcare), and separated from the tag by cleavage with rhinovirus 3C protease. His-tagged proteins were isolated on HisTrap FF columns (GE Healthcare) and eluted with an imidazole gradient. Proteins were further purified on a HiTrapQ HP column (GE Healthcare). All proteins were finally purified by size-exclusion chromatography (Superdex S75; GE Healthcare). The purification buffer was 50 mM HEPES (pH 7.2), 300 mM NaCl, 5% glycerol, and 0.5 mM tris(2-carboxyethyl)phosphine (TCEP). Mutations were generated in the constructs MLL4(5308–5537) and MLL1(3745–3969) using the Quikchange PCR mutagenesis method (Agilent).

The WRAD complex was prepared by coexpression in insect cells. Modified pFBDM and pUCDM vectors were a kind gift of Z. Zhang and D. Barford. Mouse cDNAs of full-length WDR5 with RbBP5 and Ash2L with DPY30 were cloned into these vectors using USER cloning (Berger et al., 2004; Frandsen et al., 2008). A double Strep-tag together with a tobacco etch virus cleavage site was introduced into the C terminus of RbBP5. The recombinant plasmids carrying WDR5-RbBP5 and Ash2L-DPY30 expression cassettes were transformed into DH10MultiBac_Cre_ cells (competent DH10MultiBac cells containing plasmid-expressing Cre recombinase). Expression cassettes of WDR5-RbBP5-Ash2L-DPY30 were then integrated into a bacmid by transposition and recombination (Berger et al., 2004). The WDR5-RbBP5-Ash2L-DPY30 (WRAD) was expressed using the baculovirus and insect cell (Sf9) systems, and purified through Strep-Tactin (Qiagen), anion-exchange (Resource Q), and gel-filtration chromatography (Superdex S200; GE Healthcare). Protein subunits in the complex were confirmed by peptide mass fingerprint.

### Crystallization

Crystals were obtained by the vapor diffusion method. A solution of MLL4(tag) protein (300 μM) containing SAH (600 μM) was crystallized in a condition consisting of 100 mM Tris-HCl (pH 8.5) and 20% ethanol, and improved through several rounds of seeding. Crystals were harvested into a cryobuffer consisting of the reservoir condition with 20% glycerol, and flash-frozen in liquid nitrogen.

### Structure Determination

Data were collected at the Diamond Light Source (Oxfordshire, UK) on station I02. The reflections were indexed using XDS (Kabsch, 2010) and reduced/scaled with programs from the CCP4i suite (Bailey, 1994). The structure was solved by molecular replacement using the PHASER package (McCoy et al., 2007) using the coordinates of human MLL1 SET domain from the binary complex with SAH (Southall et al., 2009). Difference maps were used to rebuild and extend the initial model using the Coot molecular graphics package (Emsley and Cowtan, 2004). Iterative cycles of refinement were carried out using REFMAC (Murshudov et al., 2011). The coordinates and structure factors for the structure have been deposited in the PDB under accession code PDB: 4Z4P.

### Methyltransferase Assays

Methyltransferase assays were performed using peptide substrates based on the histone H3 amino terminal sequence (ARTKQTARKSTGGKAPR-Y) (Cancer Research UK) either unmodified, monomethylated, or dimethylated in the underlined position. For end-point assays, reagent concentrations were 1 mM peptide, 0.5 mM SAM (including 0.625 μM ^3^H SAM [PerkinElmer]), in an assay buffer of 50 mM HEPES (pH 8), 200 mM NaCl, and 0.5 mM TCEP. Following separation of the peptide from cofactor by C18 cartridge purification (Waters), the incorporation of ^3^H-labeled SAM into the peptide was estimated by scintillation counting, as previously described (Xiao et al., 2003a). Assays were carried out at 30°C for 60 min with a final enzyme concentration of 10 μM unless otherwise stated. For kinetic analysis, the final SAM concentration was 1 mM and the peptide concentration was as stated. For kinetic analysis, raw dpm (disintegrations per minute) scintillation counter data were converted to nmol CH_3_/min/μmol enzyme, based on the assumption that 1 dpm is equivalent to 3 × 10^−14^ mmol CH_3_. This conversion assumes a radiolabel stock chemical concentration of 36.6 μM and that there is complete recovery of labeled peptide. All assays were carried out in triplicate and expressed as mean ± SD. Kinetic analysis of reaction rates was performed using the GraphPad Prism software package (GraphPad).

MALDI-TOF mass spectrometry was used to examine the reaction products of the methyltransferase reaction with the H3 peptide at specific time points, essentially as described by Guitot et al. (2014). The reaction mixture contained 10 μM enzyme (either MLL4(WIN) or MLL4(WIN) + WRAD), 200 μM SAM, and 10 μM unmodified H3 peptide in the reaction buffer described above, and was incubated at 30°C. At different time points a 10-μl aliquot of the reaction was removed and quenched by the addition of an equal volume of 1% trifluoroacetic acid, and then mixed in a 1:5 ratio with α-cyano-4-hydroxycinnamic acid. The samples were analyzed on a Bruker AutoFlex mass spectrometer (Bruker) in reflectron mode. The reactions were performed in triplicate, and the proportion of methyl species at each time point calculated by combining these multiple measurements.

Gel assays were performed in a buffer containing 40 mM HEPES (pH 8.0), 150 mM NaCl, and 2 mM DTT. Final reagent concentrations were 0.5 μM SET domain construct (containing WIN motif), 1 μM WRAD, 100 μM SAM, and either 10 μM peptide or recombinant mononucleosome substrate (prepared by the salt dialysis method [Luger et al., 1999]). Reactions were incubated at 30°C and stopped by addition of SDS sample buffer at the times indicated. Following SDS-PAGE, protein was transferred to Immobilon-P^SQ^ transfer membrane (Merck Millipore). Membrane was probed with histone H3K4 methyl-specific antibodies H3K4me1 (ab8895), H3K4me2 (ab7766), and H3K4me3 (ab8580) (Abcam) at 1:2,500 dilution in Tris-buffered saline buffer with 0.05% Tween. The blocking solution was 5% milk solution. The secondary antibody was goat antirabbit conjugated to horseradish peroxidase at 1:500 dilution (Promega), and detected by autoradiography following incubation with ECL reagent (Thermo Scientific).

### Biolayer Interferometry

The binding of the WRAD complex to MLL1 and MLL4 SET domain constructs was measured using an Octet RED biolayer interferometer (Pall ForteBio). Site-specific biotinylation of MLL1 and MLL4 constructs was achieved by the addition of an AviTag (GLNDIFEAQKIEWHE) coding sequence N-terminal to the WIN motif. Biotinylated MLL1 or MLL4 protein was immobilized onto the streptavidin biosensor (Pall ForteBio) surfaces for 5–15 min until a response of ∼1.3 nm was achieved. Binding of WRAD was measured at room temperature at concentrations in the range 62.5–500 nM by association and dissociation steps of 400 and 2000 s, respectively. The buffer used in the analysis was 50 mM Tris-HCl (pH 7.5), 350 mM NaCl, 10% glycerol, 0.5 mM DTT, and 0.05% Tween 20. Association and dissociation rate constants *k*_on_ and *k*_off_ were determined from the analysis of association and dissociation phases, respectively.

## Figures and Tables

**Table 1:** Crystallographic Data Collection and Refinement

	PDB: 4Z4P
**Data Collection**	

Space group	P2_1_
Cell Dimensions	
*a*, *b*, *c* (Å)	38.3, 40.7, 51.0
α, β, γ (°)	90.0, 91.8, 90
Resolution (Å)	2.20
*R*_merge_	0.063 (0.21)^a^
Mean *I*/σ*I*	17.3 (8.3)^a^
Completeness (%)	98.9 (98.5)^a^
Redundancy	5.4 (5.5)^a^
Wilson B factor	30.3

**Refinement**	

Resolution (Å)	2.20
No. of reflections	11,491
*R*_work_^b^/*R*_free_^c^	18.6/24.0
No. of atoms	
Total	1,422
Protein	1,361
Ligand/ion	1
Water	60
B factors (Å^2^)	
Protein	36.5
Ligand	36.6
Water	33.4
Root-mean-square deviations	
Bond lengths (Å)	0.01
Bond angles (°)	1.17

**Structure Validation (%)**	

Ramachandran outliers	0.6
Ramachandran favored	94.3
Rotamer outliers	0

a The average value is across the resolution range while that in parentheses is the value for the highest-resolution bin (2.3–2.2 Å).

b *R*_work_ = Σ | |*F*_o_| − |*F*_c_| |/Σ |*F*_o_|.

c *R*_free_ = Σ_T_ | |*F*_o_| − |*F*_c_| |/Σ_T_ |*F*_o_|, where T is a test dataset of 5% of the total reflections randomly chosen and set aside before refinement.

## Supplemental Information

Document S1. Supplemental Figures S1–S5

Document S2. Article plus Supplemental Information
