# Supplementary material for: Evolving Catalytic Properties of the MLL Family SET Domain
Source: Structure. 2015 Oct 6;23(10):1921–33. doi: 10.1016/j.str.2015.07.018 (PMC4597100; doi:10.1016/j.str.2015.07.018)
Supplement: Document S1. Supplemental Figures S1–S5 [file mmc1.pdf]

Structure, Volume *23*

Supplemental Information

Evolving Catalytic Properties of the MLL Family SET Domain

Ying Zhang, Anshumali Mittal, James Reid, Stephanie Reich, Steven J. Gamblin, and Jon R. Wilson

Evolving catalytic properties of the MLL family SET domain

Ying Zhang[1], Anshumali Mittal[1], James Reid[2,] Stephanie Reich[2], Steven J. Gamblin[1] and Jon R Wilson[1,*]

[1]The Francis Crick Institute, Mill Hill Laboratory, London, UK, NW7 1AA

[2]Domainex, Cambridge, UK, CB4 0GH

*Contact: jon.wilson@crick.ac.uk

Supplemental Data

S1: Sequence alignment of the MLL SET domains.

S2: Establishing reaction conditions for *in vitro* methyltransferase assays.

S3: Effect of the C-terminal tag on the structure of MLL4.

S4: Packing of the SET-I channel tetrapeptide.

S5: Mass Spectrometry Analysis of MLL4 Reaction Products.

S1: Related to Figure 1. Sequence alignment of the MLL SET domains.

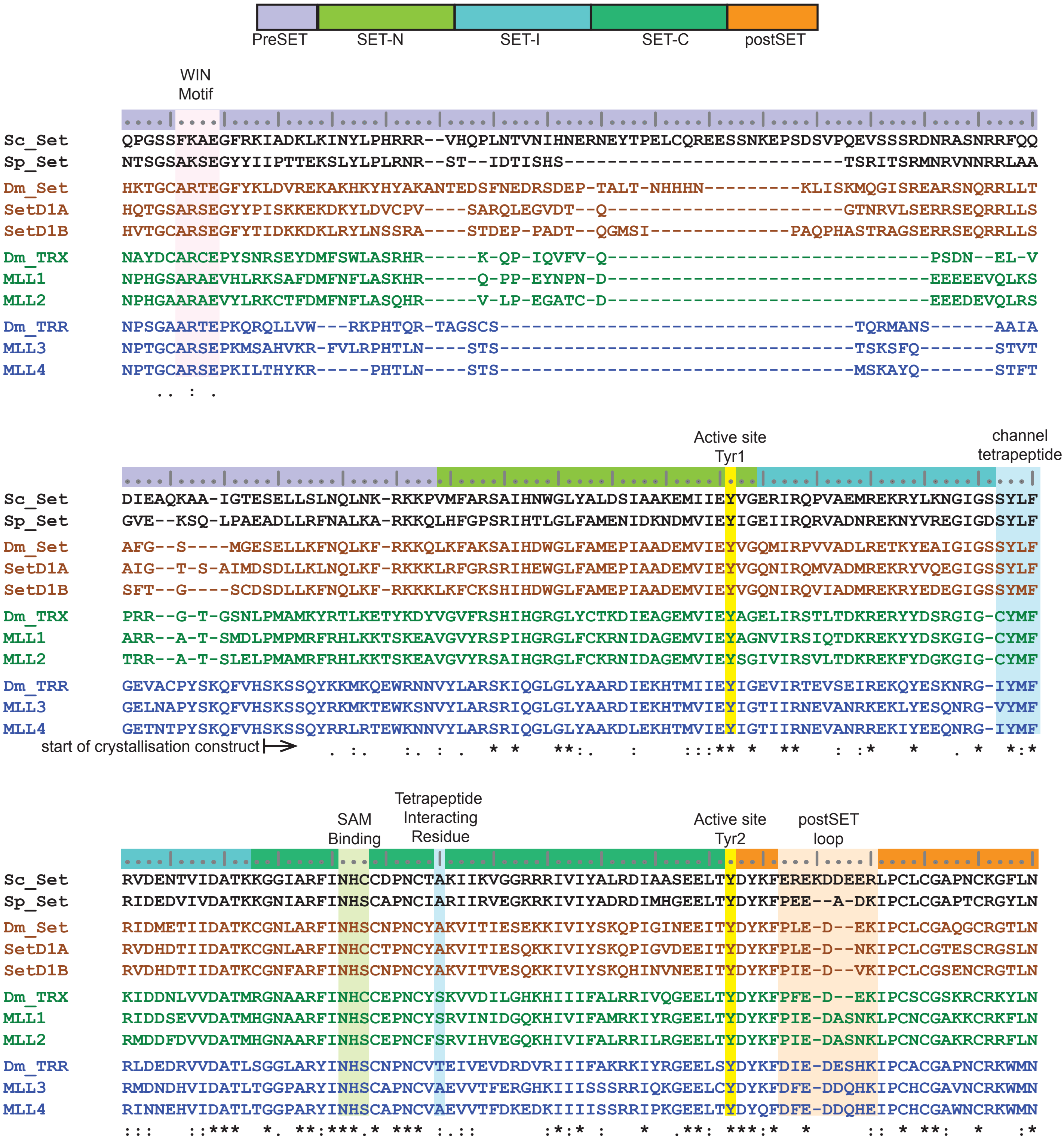

Multiple sequence alignment showing the relationship between the six human MLL SET domains, and the related proteins from *Drosophila* and yeast. The diffrerent regions of the SET domain are colored as indicated in the schematic above the alignment. The key residue positions discussed in the main text are indicated by block colors and labelled above the alignment. The start of the MLL4 crystallisation construct is indicated beneath the sequence.

The sequence alignment iillustrates the overall conservation of the SET domain, but additionally local areas of divergence that lead to the segregation into three subgroups are apparent.

S2: Related to Figure 2. Establishing reaction conditions for in vitro methyltransferase assays

A series of preliminary assays were performed in order to establish a set of methyltransferase reaction conditions, applicable to both MLL1 and MLL4 constructs, in order to compare the methyltransferase activity of recombinant SET domain constructs with peptide substrate (Figure S2). The aim was to ensure that the selected conditions were in the linear range for both enzymes whilst obtaining a reasonable signal for MLL1.

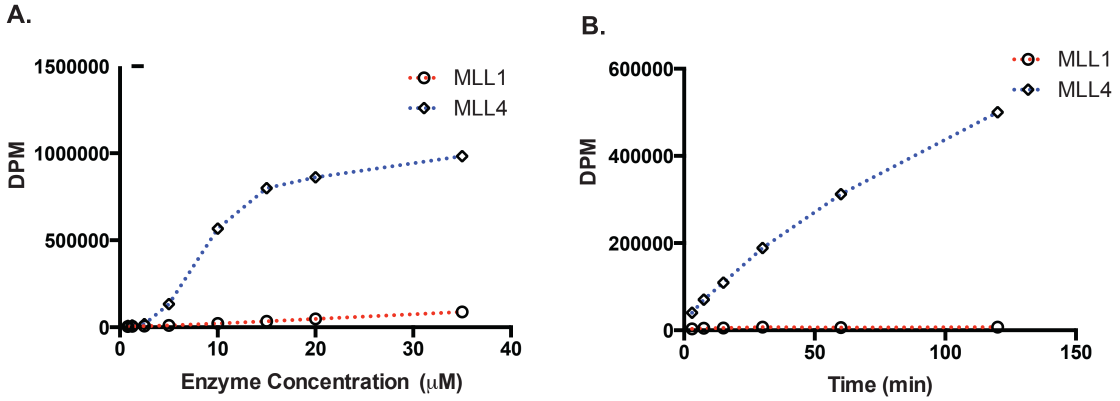

Figure S2. Selection of reaction conditions for methyltransferase assays. (A) Effect of enzyme concentration on reaction. (B) Time course of methyltransferase reaction with MLL1 and MLL4 with peptide substrate.

For endpoint assays, reagent concentrations were; 1 mM peptide, 0.5 mM SAM (including 0.625 μM ^{3}H SAM), in an assay buffer of 50 mM HEPES pH 8, 200 mM NaCl, 0.5mM TCEP. Final assays were carried out at 30°C for 60 min with an enzyme concentration of 10 μM.

All methyltransferase measurements were made in triplicate and the standard deviation indicated by the error bars. The data was normalised to the methyltransferase activity for the wild-type protein in each experiment. For the kinetic analysis (Figure 2A and B), raw scintillation values (DPM) were converted to nmoles CH_3 / min / μmol enzyme, based on the assumption that 1DPM is equivalent to 3 x 10^{-14} mmoles CH_3.

S3: Related to Figure 3. Effect of the C-terminal tag on the structure of the MLL4 SET domain

In order to obtain an MLL4 SET domain construct that yielded diffraction quality crystals a combinatorial domain hunting strategy was employed. For this a short 6xHis tag was placed at the C-terminus of the domain and N-terminal truncation constructs generated and assessed for expression and solubility (Figure S3-1).

```
                             5380                                5530
                    .........|......                    .........|.......
Native sequence     ETNTPYSKQFVHSKSS.......     ........HKIPCHCGAWNCRKWMN
Tagged construct              MTLHSKSS.......     ........HKIPCHCGAWNCRKWMKGHHHHHH

                        N-term                                       C-term
```

Figure S3-1. Sequence introduced at the N- and C-terminus of the tagged construct.

A well-behaved construct starting at residue His 5382 successfully yielded diffracting crystals, and the subsequent structure is described in the main manuscript. However, when tested in the methyltransferase assay this C-terminally tagged construct exhibited negligible activity even when compared to MLL1 (Figure S3-2). An equivalent construct but expressed with a cleavable tag at the N-terminus, leaving the C-term native, had high enzyme activity, but despite strenuous efforts did not yield diffracting crystals.

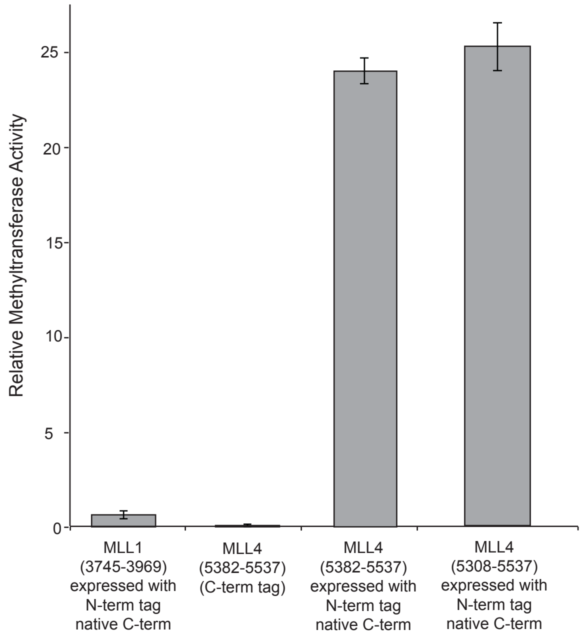

Figure S3-2. Methyltransferase assay comparing MLL1, MLL4 with a C-terminal tag, and MLL4 constructs expressed with an N-terminal tag.

The biochemical experiments presented in this manuscript describing the activity of the MLL4 SET domain were performed with MLL4 (5308-5537), which was also expressed with an N-terminal tag. This construct is slightly

longer and incorporates the conserved WIN motif known to be required for WDR5 binding, and had essentially the same activity as the MLL4 (5382-5537) construct (Figure S3-2). What impact does the tag have on the structure, and can the compromised activity be explained by the structure?

The introduced tag consists of eight residues KGHHHHHH, with the initial Lys replacing the native MLL4 C-terminal Asn residue (Figure S3-1). Using the MLL1 structure containing an intact native C-terminus as a reference, the protein would naturally terminate immediately following the conserved Cys_4Zn cluster, and make no contact with either the peptide binding groove or cofactor binding site (Figure S3-3). In the MLL4 structure, following the Cys_4Zn cluster, the tag extension makes a turn at the penultimate Met5536 residue and passes over the SAH cofactor (Figure S3-3).

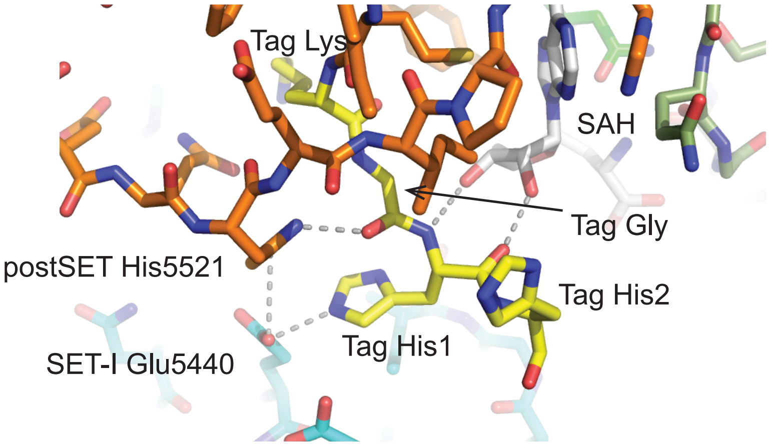

Figure S3-3. Interactions with the SET domain made by the C-terminal tag

Following the initial four tag residues, KGHH, there is a dramatic drop-off in electron density (Figure S3-4), and only these residues could be built into the model unambiguously. This implies that the rest of the tag is flexible. There are features in the density that could correspond to partial occupancy by the remaining histidine imidazole rings, indicated in Figure S3-4. Three of these features cluster around the SAH cofactor, whist a fourth density feature is adjacent to residues Trp5395 and Ile5427 in the SET-N region of the adjacent symmetry related molecule. The main chain path linking these features is not sufficiently clear to build.

The initial tag residues interact with several features on the SET domain. Firstly the Lys points towards the solvent filled cavity between the postSET and SET-I region, and the high b-factors of this side chain indicate that it is flexible. When overlapped with the MLL1 ternary structure, the tag Lys side chain could clash with the histone H3 Gln5 residue and the first tag His with the implied position of the histone H3 Ala1 (Figure S3-3).

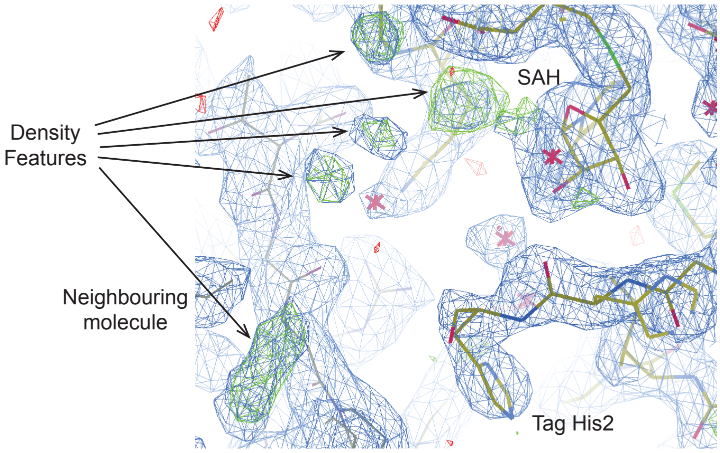

Figure S3-4. Density map of the C-terminal tag region

The main chain amide of the Gly forms a hydrogen bond with the His5521 side chain of the postSET loop. The first tag His side chain also interacts with this postSET loop residue and with the side chain of the Glu5440 residue, which is located on the SET-I helix. It cannot formally be ruled out that this interaction may influence the relative position of SET-I observed in the in MLL4 structure compared to MLL1. However the inferred flexibility of the tag, from the relatively high B-factors poor electron density, would support the hypothesis that the position of the SET-I determines that of the tag.

The second tag His side chain is orientated towards the solvent, and makes a solvent mediated interaction with the Tyr5472 hydroxyl of the neighbouring molecule, which suggests that the tag has a role in the formation of the crystal lattice.

The main chain amide and carbonyl of the first tag His make hydrogen bonds to the cofactor SAH O3’ and O2’ atoms respectively. A further water mediated hydrogen bond is made with the SAH O3’ atom and the tag Lys carbonyl. These, in addition with the density features that may arise from the less ordered tag residues surrounding the cofactor, may compromise cofactor product release. The inactivity of the MLL4-His tagged construct is therefore likely to be the combination of two features, the partial obstruction of the substrate binding grove and an extended set of interactions with the cofactor.

S4: Related to Figure 4. Packing interaction of the SET-I channel tetrapeptide

A.

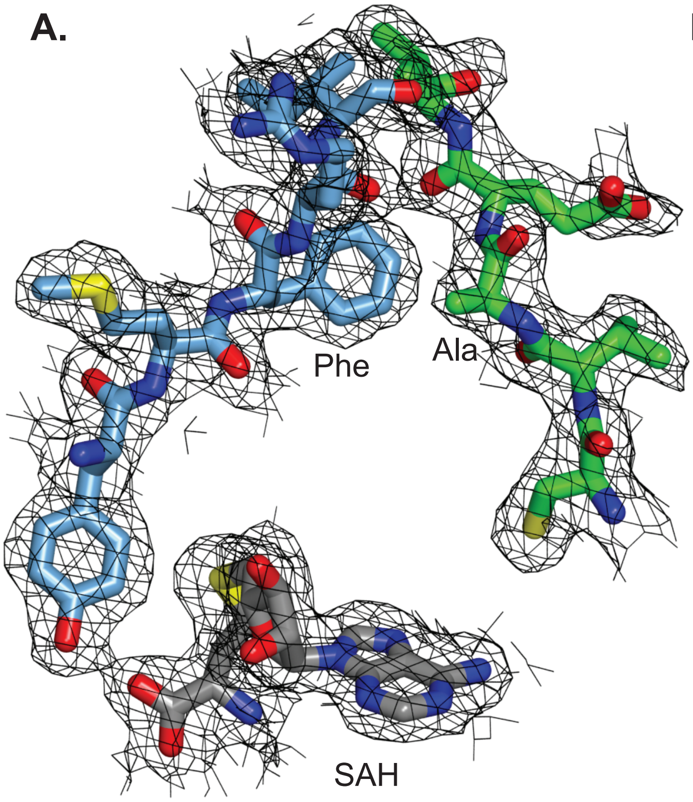

B.

	SET-I	SET-C
MLL4	Phe	Ala
MLL1	Phe	Ser
Dim5	Phe	Ile
Suv39	Phe	Val
EzH2	Phe	Ala
G9a	Phe	Pro
Set8	Tyr	Thr
Set7	Tyr	Ile

The SET-I region of MLL1 SET domain adopts an atypical position that creates an unusually ‘open’ active site that is less optimal for methyl transfer. The position of the SET-I in MLL4 is more conventional. The region of the SET-I that packs against the target lysine residue is the channel peptide. To enclose the active site, the channel tetrapeptide packs against the first strand of the SET-C region. Comparing the MLL1 and MLL4 structures reveals that this interaction is less favourable for MLL1.

(A). Electron density map (2Fo-Fc) of the MLL4 channel tetrapeptide and first SET-C strand showing how the SET-I phenylalanine interacts with the Set-C alanine and creates the active site.

(B). List of the equivalent Set-I and Set-C residues from a range of SET domains. Only the MLL1 SET domain has a sequence mismatch, phenylaanine to serine, that generates a less favourable packing interaction.

S5: Related to Figure 6. Mass Spectrometry Analysis of MLL4 Reaction Products

Mass spectrometry was used to determine the methylation state of the products of the MLL4 reaction. Unmodified H3 peptide substrate was incubated with an equimolar concentration of MLL4 or MLL4 + WRAD with excess SAM cofactor. After the indicated time points the reaction was stopped by the addition of TFA buffer. The reaction mixture was analysed using matrix-assisted laser desorption/ionisation time-of-flight mass spectrometry*. Example profiles for the indicated time points are presented below.

Under these conditions, for MLL4 alone, initially only H3K4me1 product was detected, but following overnight incubation the H3K4me2 species was also observed. In the presence of WRAD the reaction proceeded more rapidly and within the first 30 min H3K4me2 predominated. Following overnight incubation the reaction proceeded to H3K4me3.

*Guitot, K., Scarabelli, S., Drujon, T., Bolbach, G., Amoura, M., Burlina, F., Jeltsch, A., Sagan, S., and Guianvarc'h, D. **(2014)**. Label-free measurement of histone lysine methyltransferases activity by matrix-assisted laser desorption/ionization time-of-flight mass spectrometry. Analytical Biochemistry ***456***, 25-31.

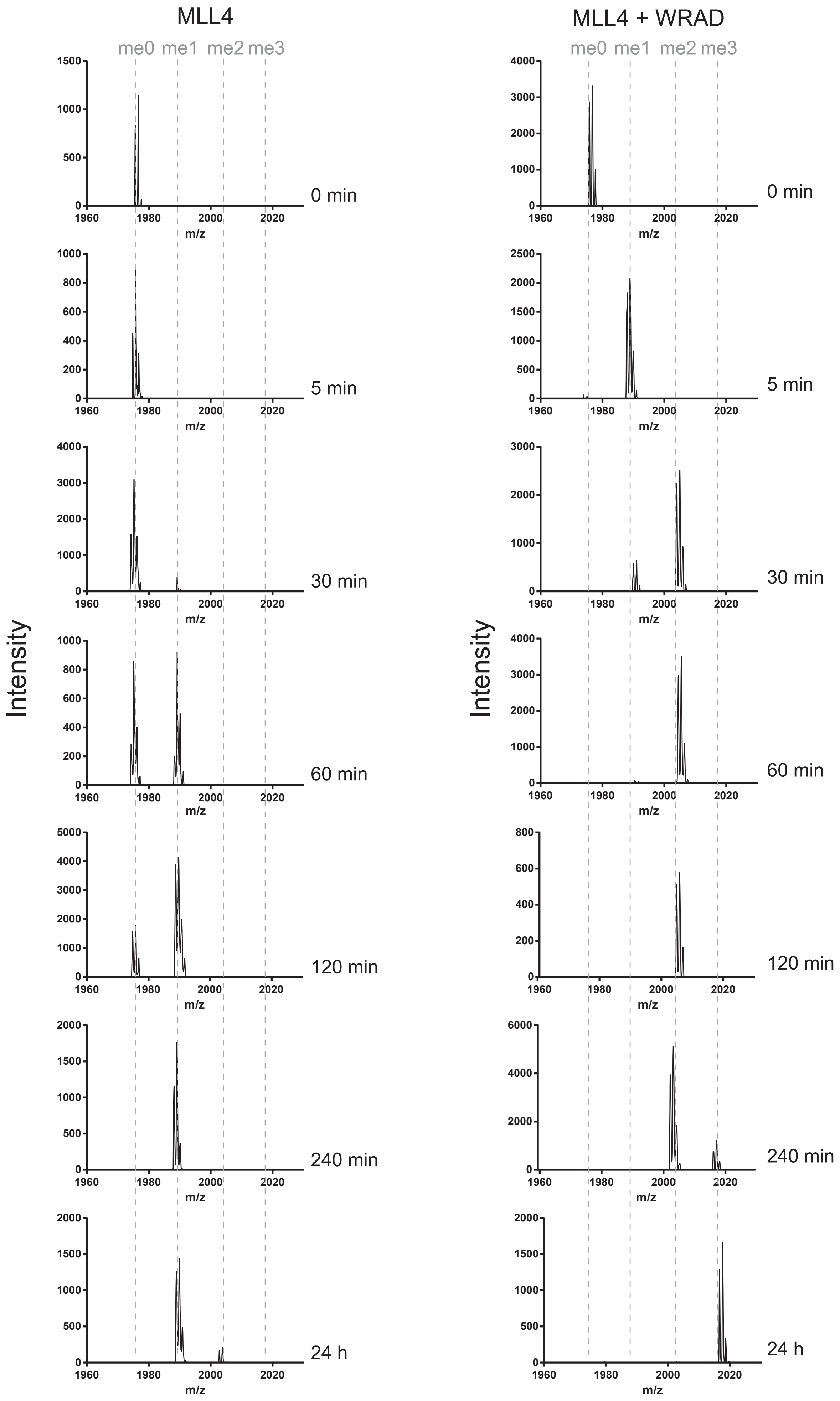

MLL4
MLL4 + WRAD
me0
me1
me2
me3
Intensity
m/z
0 min
5 min
30 min
60 min
120 min
240 min
24 h
